# Supplementary material for: Treatment outcome of Severe Acute Malnutrition and associated factors among under-five children in outpatient therapeutics unit in Gubalafto Wereda, North Wollo Zone, Ethiopia, 2019
Source: PLoS One. 2020 Sep 3;15(9):e0238231. doi: 10.1371/journal.pone.0238231 (PMC7470268; doi:10.1371/journal.pone.0238231)
Supplement: Attachment — Submitted filename: PONE-D-19-29446_reviewer (1).pdf [file pone.0238231.s001.pdf]

PLOS ONE

Treatment Outcome of Severe Acute Malnutrition and associated factors among under-five children in outpatient therapeutics unit in Gubalafto Wereda, North Wollo Zone, Amara Regional State, North Ethiopia, 2019 G.C

--Manuscript Draft--

**Manuscript Number:**	PONE-D-19-29446
**Article Type:**	Research Article
**Full Title:**	Treatment Outcome of Severe Acute Malnutrition and associated factors among under-five children in outpatient therapeutics unit in Gubalafto Wereda, North Wollo Zone, Amara Regional State, North Ethiopia, 2019 G.C
**Short Title:**	Treatment Outcome of Severe Acute Malnutrition and associated factors
**Corresponding Author:**	Biruk Abate Beletew, MSc<br>Woldia University<br>Woldia, Amhara ETHIOPIA
**Keywords:**	Ethiopia, Gubalafto, Outpatient Therapeutic Program, Severe Acute Malnutrition, treatment outcome, Ethiopia
**Abstract:**	Background<br><br>In Ethiopia uncomplicated severe acute malnutrition is managed through the outpatient therapeutic program at health posts level. This brings the services for the management of Severe Acute Malnutrition closer to the community by making services available at decentralized treatment points within the primary health care settings. So far, evidence on the treatment outcome of the program is limited. Thus, the main aim of this study was to determine the magnitude of treatment outcomes of severe acute malnutrition and associated factors among under-five children at outpatient therapeutic feeding units.Methods<br><br>This was a retrospective cohort study conducted on 600 children who had been managed for SAM under OTP in Gubalafto Wereda from April to May/2019. The children were selected using systematic random sampling from 9 health posts. The structured, pre-tested and adapted questionnaire was used to collect the data. The data was entered by using EPI-data Version 4.2 and exported to SPSS version 24.0 for analysis. Bivariate and Multivariate regression was also carried out to determine the association between dependent and independent variables.Results<br><br>A total of 600 records of children with a diagnosis of severe acute malnutrition were reviewed. Of these cases of malnutrition, the recovery rate was revealed as 65%. The death rate, default rate, and medical transfer were 2.0, 16.0, and 17.0 respectively. Children who took immunization were had 6.85 times higher odds of recovery than children who were not immunized (AOR=6.85 at 95% CI (3.68-12.76)). The likelihood of recovery was 3.78 times higher among children with new admission than those with re-admission (AOR=3.78at 95% CI ((1.77-8.07))). Likewise, children provided with amoxicillin were 3.38 times more likely to recover compared to their counterparts who were not provided (AOR=3.38 at 95% CI ((1.61-7.08))).Conclusions<br><br>The recovery rate and medical transfer were lower than sphere standard. Presence of cough, presence of diarrhea admission category, provision of amoxicillin, and immunization status were factors identified as significantly associated with treatment outcome of Sever Acute Malnutrition. Building capacity of OTP service providers and regular monitoring of service provision based on the management protocol were recommended.
**Order of Authors:**	Biruk Abate Beletew, MSc
	Befkad Deresse
	Ayelign Mengesha
	Mesfin Wudu

Powered by Editorial Manager® and ProduXion Manager® from Aries Systems Corporation

Additional Information:	
**Question**	**Response**
**Financial Disclosure**<br><br>Enter a financial disclosure statement that describes the sources of funding for the work included in this submission. Review the submission guidelines for detailed requirements. View published research articles from *PLOS ONE* for specific examples.<br><br>This statement is required for submission and **will appear in the published article** if the submission is accepted. Please make sure it is accurate.<br><br>**Unfunded studies**<br>Enter: *The author(s) received no specific funding for this work.*<br><br>**Funded studies**<br>Enter a statement with the following details:<br>• Initials of the authors who received each award<br>• Grant numbers awarded to each author<br>• The full name of each funder<br>• URL of each funder website<br>• Did the sponsors or funders play any role in the study design, data collection and analysis, decision to publish, or preparation of the manuscript?<br>• **NO** - Include this sentence at the end of your statement: *The funders had no role in study design, data collection and analysis, decision to publish, or preparation of the manuscript.*<br>• **YES** - Specify the role(s) played.<br><br>* typeset	N/A
**Competing Interests**<br><br>Use the instructions below to enter a competing interest statement for this submission. On behalf of all authors, disclose any competing interests that could be perceived to bias this work—acknowledging all financial support and any other relevant financial or non-financial competing interests.	The authors declare that they have no competing interests

This statement **will appear in the published article** if the submission is accepted. Please make sure it is accurate. View published research articles from *PLOS ONE* for specific examples.<br><br>**NO authors have competing interests**<br><br>Enter: *The authors have declared that no competing interests exist.*<br><br>**Authors with competing interests**<br><br>Enter competing interest details beginning with this statement:<br><br>*I have read the journal's policy and the authors of this manuscript have the following competing interests: [insert competing interests here]*<br><br>* typeset	
**Ethics Statement**<br><br>Enter an ethics statement for this submission. This statement is required if the study involved:<br><br>• Human participants<br>• Human specimens or tissue<br>• Vertebrate animals or cephalopods<br>• Vertebrate embryos or tissues<br>• Field research<br><br>Write "N/A" if the submission does not require an ethics statement.<br><br>General guidance is provided below. Consult the submission guidelines for detailed instructions. **Make sure that all information entered here is included in the Methods section of the manuscript.**	Ethics approval and consent to participate<br>Ethical approval was obtained from the research ethics review board of the WU faculty of health science. An official letter of permission was obtained from WU faculty of health science and was submitted to the respective administrative bodies of the Gubalafto Woreda; permission from these administrative bodies was also given. Confidentiality was ensured throughout the research process. All incomplete charts were considered as non-response rate

Powered by Editorial Manager® and ProduXion Manager® from Aries Systems Corporation

**Format for specific study types**<br><br>**Human Subject Research (involving human participants and/or tissue)**<br>• Give the name of the institutional review board or ethics committee that approved the study<br>• Include the approval number and/or a statement indicating approval of this research<br>• Indicate the form of consent obtained (written/oral) or the reason that consent was not obtained (e.g. the data were analyzed anonymously)<br><br>**Animal Research (involving vertebrate animals, embryos or tissues)**<br>• Provide the name of the Institutional Animal Care and Use Committee (IACUC) or other relevant ethics board that reviewed the study protocol, and indicate whether they approved this research or granted a formal waiver of ethical approval<br>• Include an approval number if one was obtained<br>• If the study involved *non-human primates*, add *additional details* about animal welfare and steps taken to ameliorate suffering<br>• If anesthesia, euthanasia, or any kind of animal sacrifice is part of the study, include briefly which substances and/or methods were applied<br><br>**Field Research**<br><br>Include the following details if this study involves the collection of plant, animal, or other materials from a natural setting:<br>• Field permit number<br>• Name of the institution or relevant body that granted permission	
**Data Availability**<br><br>Authors are required to make all data underlying the findings described fully available, without restriction, and from the time of publication. PLOS allows rare exceptions to address legal and ethical concerns. See the PLOS Data Policy and FAQ for detailed information.	Yes - all data are fully available without restriction

A Data Availability Statement describing where the data can be found is required at submission. Your answers to this question constitute the Data Availability Statement and **will be published in the article**, if accepted.<br><br>**Important:** Stating 'data available on request from the author' is not sufficient. If your data are only available upon request, select 'No' for the first question and explain your exceptional situation in the text box.<br><br>Do the authors confirm that all data underlying the findings described in their manuscript are fully available without restriction?	
**Describe where the data may be found in full sentences. If you are copying our sample text, replace any instances of XXX with the appropriate details.**<br><br>• If the data are **held or will be held in a public repository**, include URLs, accession numbers or DOIs. If this information will only be available after acceptance, indicate this by ticking the box below. For example: *All XXX files are available from the XXX database (accession number(s) XXX, XXX.*).<br>• If the data are all contained **within the manuscript and/or Supporting Information files**, enter the following: *All relevant data are within the manuscript and its Supporting Information files.*<br>• If neither of these applies but you are able to provide **details of access elsewhere**, with or without limitations, please do so. For example:<br><br>*Data cannot be shared publicly because of [XXX]. Data are available from the XXX Institutional Data Access / Ethics Committee (contact via XXX) for researchers who meet the criteria for access to confidential data.*<br><br>*The data underlying the results presented in the study are available from (include the name of the third party*	NO

and contact information or URL).<br>• This text is appropriate if the data are owned by a third party and authors do not have permission to share the data.<br><br>* typeset	
Additional data availability information:	Tick here if the URLs/accession numbers/DOIs will be available only after acceptance of the manuscript for publication so that we can ensure their inclusion before publication.; Tick here if your circumstances are not covered by the questions above and you need the journal’s help to make your data available.

Click here to download Manuscript final ffffMANSCRPIT SAM TREATMENT OUTCOM.edited.docx

Treatment Outcome of Severe Acute Malnutrition and associated factors among under-five children in outpatient therapeutics unit in Gubalafto Wereda, North Wollo Zone, Amara Regional State, North Ethiopia, 2019 G.C

Biruk Beletew, Befkad Deresse, Ayelign Mengesha, Mesfin Wudu

Authors:

[1*]Biruk Beletew (MSc in pediatrics & child health nursing)

[1]Woldia University, Faculty of Health Sciences, Department of Nursing, P.O.Box 400 Woldia, Ethiopia:

E-mail: birukkelemb@gmail.com ; ***Tel***: +251922898070

Befkad Adresse(BSc in Nursing)

[1]Woldia University, Faculty of Health Sciences, Department of Nursing, P.O.Box 400 Woldia, Ethiopia

E-mail: addis23r@gmail.com

Ayelign Mengesha (MSc in adult health nursing)

[1]Woldia University, Faculty of Health Sciences, Department of Nursing, P.O.Box 400 Woldia, Ethiopia:

E-mail: Ayelignmengesha59@gmail.com ; ***Tel***: +251937384459

Mesfin Wudu (MSc in pediatrics & child health)

[1]Woldia University, Faculty of Health Sciences, Department of Nursing, P.O.Box 400 Woldia, Ethiopia:

E-mail: mesfine12a@gmail.com; ***Tel***:+251962649062

****Corresponding Author*: Biruk Beletew, E**-*mail*: birukkelemb@gmail.com

Running title: Treatment outcome of severe acute malnutrition

ABSTRACT

Background: In Ethiopia uncomplicated severe acute malnutrition is managed through the outpatient therapeutic program at health posts level. This brings the services for the management of Severe Acute Malnutrition closer to the community by making services available at decentralized treatment points within the primary health care settings. So far, evidence on the treatment outcome of the program is limited. Thus**, t**he main aim of this study was to determine

the magnitude of treatment outcomes of severe acute malnutrition and associated factors among under-five children at outpatient therapeutic feeding units.

Methods: This was a retrospective cohort study conducted on 600 children who had been managed for SAM under OTP in Gubalafto Wereda from April to May/2019. The children were selected using systematic random sampling from 9 health posts. The structured, pre-tested and adapted questionnaire was used to collect the data. The data was entered by using EPI-data Version 4.2 and exported to SPSS version 24.0 for analysis. Bivariate and Multivariate regression was also carried out to determine the association between dependent and independent variables.

Results: A total of 600 records of children with a diagnosis of severe acute malnutrition were reviewed. Of these cases of malnutrition, the recovery rate was revealed as 65 %. The death rate, default rate, and medical transfer were 2.0, 16.0, and 17.0 respectively. Children who took immunization were had 6.85 times higher odds of recovery than children who were not immunized (AOR=6.85 at 95% CI (3.68-12.76)). The likelihood of recovery was 3.78 times higher among children with new admission than those with re-admission (AOR=3.78at 95% CI ((1.77-8.07))). Likewise, children provided with amoxicillin were 3.38 times more likely to recover compared to their counterparts who were not provided (AOR=3.38 at 95% CI ((1.61-7.08))).

Conclusions: The recovery rate and medical transfer were lower than sphere standard. Presence of cough, presence of diarrhea admission category, provision of amoxicillin, and immunization status were factors identified as significantly associated with treatment outcome of Sever Acute Malnutrition. Building capacity of OTP service providers and regular monitoring of service provision based on the management protocol were recommended.

Keywords: Ethiopia, Gubalafto, Outpatient Therapeutic Program, Severe Acute Malnutrition, treatment outcome, Ethiopia

Background

Malnutrition is a condition that results from deficiencies, excess or imbalance in a person's intake of energy or nutrients. It is broadly classified as undernutrition includes stunting, wasting, underweight and micronutrient deficiency and the other form is overweight and obesity(1).

Formerly in many countries, treatment of SAM had been restricted to facility-based approaches, greatly limiting its coverage and impact. However, evidence from emergency programs suggested that large numbers of SAM cases could be treated in their communities without being admitted to a health facility. The program was started in Ethiopia in 2003 using the CTC model, which depended upon significant external resources and expertise and was implemented parallel to the national health system rather than integrated(4, 5).This community-based management of acute malnutrition (CMAM) reduce limitations of health facilities and therapeutic feeding centers (TFC) management of SAM .It addresses community based management of SAM children without medical complications (OTP) and MAM and designed as components of CMAM(1, 3, 6).

In Ethiopia, the program now expanded to every health center and health post of the country. OTP serve the management of SAM in children aged 6– 59 months (4, 7). The management of SAM was mainly with ready-to-use therapeutic foods (RUTF); other routine medications like antibiotics, vitamin A, and folic acid; and deworming(7, 8).

Globally, 52 million children of age less than five years were affected by acute malnutrition from which 17 million were severely wasted. Data shows that more than half of all wasted children in the world live in Southern Asia and Sub-Sahara African countries. Undernutrition is also a major cause of disability preventing children who survive from reaching their full development potentials(10, 11)

Severe acute malnutrition is still a major public health problem in many African countries affecting the overall health and development priorities due to the effects(11, 14).

Despite the improvement made in child health and nutritional interventions, Ethiopia remains in a precarious situation where undernutrition is an underlying cause to half of its child deaths and wasting contributing to 23% of these deaths(15).

In Ethiopia, studies indicate that the recovery rate among children attending the inpatient facilities was still low and the defaulter rate was high compared to the acceptable minimum standard, this has the negative impact on the child health and survival(16, 17).

Ethiopia Demographic and Health Survey (EDHS) 2016 report showed that 38%, 10% and 24% of under 5 years of age children in Ethiopia were stunted, wasted and underweight respectively. In Amara region, 11.6% of under-five years of age children were wasted of which 3.5 are severely wasted (3).

Inpatient therapeutic feeding units are faced with a lot of challenges in handling cases of severe acute malnutrition. Some of the challenges include; limited in-patient capacity, lack of enough skilled staff in the hospitals to treat the large numbers needing care, the centralized nature of hospitals promotes late presentations and high opportunity cost for careers, serious risk of cross infections for immune-suppressed children such as children with SAM(18).

Besides the prevention strategies, the improved management of SAM is an integral part of the World Health Resolution on Infant and Young Child Nutrition to improve child survival. Children with SAM have profoundly disturbed physiology and metabolism, such that if intensive refeeding is initiated before metabolic and electrolyte imbalances corrected(9).

Despite malnutrition is one of the major public health problems in Ethiopia, limited information exists regarding the outcome of SAM treatment provided through the outpatient decentralized approach. Besides, the high percentage of malnutrition is alarming which needs further study to describe the treatment outcome of SAM in OTP to assess the factors contributing to the treatment outcome. The study, therefore, is aimed at describing the treatment outcome among children of age less than five years and identifies factors contributing to the treatment outcome.

Methods

Study area and periods

The study was conducted in GubalaftoWereda from April 2016 to May 2019 GC. Gubalafto is one of the Wereda in North Wollo Zone of Amhara Region which is found at 521 km far from Addis Ababa, North-Central Ethiopia. The total estimated population in the Woreda is 176492 of whom, 90,187 male and 86305 females. The number of under-five children in this Wereda is 23,904. The Woreda has a total 34 kebeles; among which 30 kebeles are rural and 4 kebeles are urban.GubalaftoWereda administration health office report, there are 8 health centers and 34 health posts(28).

Study design

A retrospective cohort study was conducted using document review in outpatient therapeutic feeding units of the selected health posts North Wollo Zone, Amhara region, Ethiopia, 2019.

Population

Source population

All under-five children at outpatient therapeutic feeding units with the diagnosis of severe acute malnutrition in North Wollo Zone health posts

Study population

Under-five children at outpatient therapeutic feeding units of the selected health posts

Study unit

Medical records of the sampled under-five children at outpatient therapeutic feeding units of the selected health posts

Eligibility Criteria

Inclusion criteria

Records of under-five children at outpatient therapeutic feeding units

Exclusion criteria

Transferred cases and records with incomplete information will exclude.

Sampling technique and procedure

Multistage sampling technique was employed to select the study subject. The study area, Gubalafto Wereda has a total of 34 Kebeles (4 are urban and 30 are rural kebeles). From the total 34 kebeles, 7 rural and 2 urban kebeles was selected by simple random sampling method.

The samples are distributed proportionally based on probability proportional to size (PPS) allocation technique. Participants in each kebele are selected by using a systematic sampling technique after calculating the sampling interval (K) for each kebeles.

The sample frame is the list of under-five SAM children charts at OTP. It is identified after checking all 9 selected kebeles (study population) to identify charts of children from birth up to 59 months old and coding of those charts was done to prepare sampling frame for each kebele. Those children with incomplete charts are considered as non-respondent. Finally, the OTP record card of each child was selected using systematic random sampling.

Sample size determination

For the first specific objective sample size for the magnitude of treatment, the outcome was determined using the sample size determination formula for a single population proportion. A study done in Wolaita zone showed a recovery rate of 64.9% and two different studies in Amhara region showed a recovery rate of 78% & 58.4%. For this calculation, we use the proportion that was done in Wolaita since the two lists above done in inpatient therapeutics unit.

$$n= \frac{(z\ \alpha/2)^2 \times p\ (1-p)}{d^2}$$

$$n= \frac{(1.96)^2 \times 0.64\ (1-0.64)}{(0.05)^2}$$

$$= 353.89$$

$$=354$$

A total sample size of 354 was determined using single sample proportion formula by considering 95% confidence, 5% margin of error and taking 64.9 % recovery rate from Wolaita. By adding 10% non- respondent rate the final sample size was 390

Where,

n = sample size derived from estimation formula

$Z\alpha/2$ = the value of z at a confidence level of 95%= 1.96

P= is recovery rates of children who had been managed for SAM = .64.9(64.9%)

d = is the margin of error to be tolerated and taken as 5%

Considering 10% contingency for missing data the final sample size for determining the treatment outcome

For the second objective, the sample size was determined using a double population proportion formula by considering study done in Tigray and Wolaita recovery rate p=61.78,64.9 respectably to calculate the required sample size. Finally, it is calculated by using Epi info version 7 statistical packages.

$$n_1 = \frac{\left[Z_{\alpha/2}\sqrt{\left(1+\frac{1}{r}\right)P(1-P)} + Z_{\beta}\sqrt{\frac{P_1(1-P_1)+P_2(1-P_2)}{r}} \right]^2}{(P_1-P_2)^2}$$

- ✓ **P1:** is a percent of exposed with the outcome
- ✓ **P2:** is a percent of non-exposed with the outcome
- ✓ **$Z_{\alpha/2}$:** is taking CI 95%,
- ✓ Z_{B}:80% of power
- ✓ And **r** is the ratio of non-exposed to exposed 1:1

Sampling procedure

We used Open Epi-version 2.3 (20) to calculate the sample size with the following assumptions: The proportion recovered in the exposed (children with Comorbidities) group (33.3%), the proportion recovered in the non-exposed (children without Comorbidities) group (20.4%)(29), 95% CI (confidence interval), 5% marginal error (d), and power of 80%. Accordingly, the minimum sample size calculated for each group was374. We used a design effect of 1.5 to compensate for potential losses during multi-stage sampling and added 10% of the sample for missing and incomplete data. The final sample size obtained was600.

The study area, Gubalafto Wereda has a total of 34 Kebeles (4 are urban and 30 are rural kebeles). From the total 34 kebeles, 7 rural and 2 urban kebeles was selected by simple random sampling method.

The samples are distributed proportionally based on probability proportional to size (PPS) allocation technique. Participants in each kebele are selected by using a systematic sampling technique after calculating the sampling interval (K=2) for each kebeles.

The sample frame is the list of under-five SAM children charts at OTP. It is identified after checking all 9 selected kebeles (study population) to identify charts of children from birth up to 59 months old and coding of those charts was done to prepare sampling frame for each kebele those children with incomplete charts are considered as non-respondent. Finally, the OTP record card of each child was selected using systematic random sampling.

Study variables

Independent variables

Socio-demographic variables

Age

Sex

Type of malnutrition

Marasmus

Kwashiorkor

Marasmic kwashiorkor

Medical co-morbidities

TB

HIV

Cough or pneumonia

Fever

Diarrhea

Immunization

Measles vaccine

Vitamin A

Routine medications

Amoxicillin

Folic acid

Albendazole or Mebendazole

Admission category

New

Readmission

Dependent variables

Treatment outcome Recovered or Not Recovered

Operational definitions

1. Treatment outcome: grouped as recovered and not recovered from SAM management at outpatient therapeutic feeding units in this study
2. Recovered: children with severe acute malnutrition declared as cured | Recovered in the logbook of outpatient therapeutic feeding units.
3. Not recovered: defined as children discharged from outpatient therapeutic feeding units with outcome other than recovery in this study (death, default, and non-responder).
4. Severe acute malnutrition (SAM): the weight-for-height ratio of less than minus 3 standard deviations below the median WHO growth standards or weight-for-height ratio of below 70% of the median NCHS reference or presence of nutritional edema.
5. Outpatient Management: Management of SAM of children without medical complications or pass appetite test.
6. Defaulter: A SAM patient who become absent continuously from the therapeutic feeding program of outpatient care.
7. Non-responder: SAM patient admitted to inpatient that do not reach discharge criteria after 40 days in the inpatient program.
8. Died: Severe Acute Malnutrition Patient in OTP as died.
9. Type of malnutrition: grouped as marasmus (non- edematous), kwashiorkor (edematous), marasmus kwashiorkor (both edema and severe wasting) and visible severe wasting.

Data quality control

The data collectors and the supervisors were trained for two days on techniques of data collection and the importance of disclosing the possible purposes of the study to the study participants before the start of data collection. To assure the quality of the data, investigators closely supervised the data collection procedure daily. The review was made in the field for checking the completeness of questionnaire and correction was made in the field.

Each questionnaire and data sheet was check before the data entry. The data was entered one I data version 4.2 daily, basis and missing data were identified. Incorrectly filled or questioners that miss major content was not included in the study. The pretest was conducted in Woldia

health center (which is not a study area) using 5% of the total sample size which is not included in the actual sampling and necessary adjustments were made on the tool.

Data processing and analysis

The data was entered and analyzed by using EPI-data Version 4.2 and exported to SPSS version 24.0 for analysis. Bivariate and Multivariate regression was also carried out to determine the association between dependent and independent variables.

Ethical consideration

Ethical approval was obtained from the research ethics review board of the WU faculty of health science. An official letter of permission was obtained from WU faculty of health science and was submitted to the respective administrative bodies of the Gubalafto woreda; permission from these administrative bodies was also given. Confidentiality was ensured throughout the research process. All incomplete charts were considered as non-response rate.

Results

Socio-demographic characteristics of children

The study included 6oo eligible children who had been managed for SAM under the OTP from April to May (2016-2019); 50.8% of children enrolled in the study were males. Children beyond two years of age, 179(29.8%), were underrepresented in the OTP as compared to their middle age groups, 313(52.2%). About 18% of the children were younger. Concerning vaccination history: 444(74.0%) were fully vaccinated, 83(13.8%) were partially vaccinated, 40(6.7%) unknown vaccination status and 33(5.5%) were not vaccinated for age. The majority (90.2%) of children was identified as newly admitted children. Regarding treatment outcome 65% recovered 2% were dead(Table 1).

Table 1: Socio-Demographics and related characteristics of children from birth up to 59 months in Gubalafto Wereda, North Wollo Zone, Amhara, Ethiopia, 2019 (N= 600)

Socio-demographic and related variables		**Frequency (N=600)**	**Percent**
**Age**	<6 month	108	18
	6-24 month	313	52.2

	>24 months	179	29.8
**Sex**	Male	305	50.8
	Female	295	49.2
**Immunization status**	[illegible]cinated for age	444	74.0
	partially vaccinated	83	13.8
	not vaccinated	33	5.5
	Unknown	40	6.7
**Season**	Winter	142	23.7
	Spring	189	31.5
	summer	167	27.8
	autumn	102	17.0
**Weight**	<=6.5	228	38.0
	>6.5	372	62.0
**Admission category**	New	540	90.2
	readmission	59	9.8
**Temperature**	<=38	491	81.8
	>38	109	18.2
**Respiratory rate**	30-40	468	78.1
	40-50	131	21.9
**Treatment outcome**	cured	390	65.0
	dead	12	2.0
	defaulter	96	16.0
	medical transfer	102	17.0

Regarding the type of Malnutrition at Admission about 451 (75.2%) of children admitted to OTP had non-edematous (marasmic), type of severe acute malnutrition at admission (visit) (149)24.8% of the children were kwashiorkor.

Co-morbidity at Admission

Forty present (40%) of children admitted to OTP had a fever. one or more comorbidities at admission such as diarrhea (12.8), HIV positive 12(2%), TB 12(2%), cough 19.2% and vomiting 25.3%. In addition to this 26.4 % of the children had edema (Table 2).

Table 2: Co-morbidity at Admission of children from birth up to 59 months in GubalaftoWereda, North Wollo Zone, Amhara, Ethiopia, 2019 (N= 600)

Characteristics	Category	Frequency	Percent (%)
**Child HIV status**	Negative	475	79.2
	Positive	12	2
	Unknown	113	18.8
**Presence of TB**	Yes	12	2
	No	482	80.3
	Unknown	106	17.7
**Presence of fever**	yes	250	41.7
	No	350	58.3
**Presence of cough**	yes	115	19.2
	No	485	80.8
**Presence diarrhea**	Yes	77	12.8
	No	523	87.2
**Presence of vomiting**	Yes	152	25.3
	No	448	74.7
**Presence of edema**	**Yes**	158	**26.4**
	No	441	73.6

Routine medications

Admitted cases with severe acute malnutrition to OTP were managed following the federal ministry of health of Ethiopia guideline protocol for the treatment of severe acute malnutrition. Out of 600 children whose medication records were available for review, the most prescribed

medications were PO antibiotics (90%) Amoxicillin followed by Vitamin A supplementation (74.%). Of the total 44.6% of the children was dewormed with Albendazole or Mebendazole, 52.2% received folic acid(Table 3).

Table 3**:** Routine medication given at OTP for SAM children from birth up to 59 months in Gubalafto Wereda, North Wollo Zone, Amhara, Ethiopia, 2019 (N= 600)

**Routine medication**		**Frequency (N=600)**	**Percent**
Antibiotic/s (PO)	Yes	540	90
	No	60	10
Vit A	Yes	444	74
	no	156	26
Folic acid	Yes	313	52.2
	No	287	47.8
Albendazole or Mebendazole	Yes	267	44.6
	No	332	55.4

Bivariate and Multivariate analysis on treatment outcome of SAM and associated factors

In bivariate logistic regression analysis type of malnutrition, presence of fever, presence diarrhea, presence cough, presence vomiting, presence edema, PO antibiotics, admission category, immunization status, the weight of child were associated with treatment outcome of SAM. Those variables that have a p-value less than or equal to 0.25 were entered to a multivariable logistic regression model to adjust for possible confounders.

In multivariate logistic regression presence of cough, presence of diarrhea PO antibiotics, admission category, the immunization status of a child be significantly associated with the treatment outcome of SAM. Accordingly, the odds of recovery on SAM among children presenting with cough [AOR = 0.47,95 % CI: (0.28-0.80)] was lower than as compared to those without cough. The odds of recovery on SAM were higher among children took PO antibiotics [AOR=3.38, 95% CI: (1.61 -7.08)] as compared to those who were not taken. Admission

category was also associated with the recovery of SAM. Those who have new admission were 3.78 times more likely to recover than those who have readmission. The odds of recovery on SAM among children presence with diarrhea[AOR = .46,95 % CI: (.25-.86)]less likely recover than those SAM children without diarrhea. Concerning immunization status of children the odds of recovery on SAM management among children who have been fully vaccinated (AOR=6.85, 95% CI: 3.68-12.78)) was higher as compared to those who have been not vaccinated(Table 4).

Table 4: Bivariate and Multivariate analysis on treatment outcome of SAM and associated factors

Variables	Treatment outcome (N=600)		Odds Ratios		P-value
	Recovered frequency (%)	Not recovered Frequency (%)	COR(95% CI)	AOR(95% CI)	
Type of malnutrition					
Marasmus	318(53.0)	133(22.2)	2.56(1.75-3.74)	1.71(.72-4.08)	.22
Kwashiorkor	72(12.0)	77(12.8)			
Presence of TB					
Yes	5(0.8%)	7(1.2%)	.34(.10-1.14)	.70(.16-2.99)	.63
No	313(52.2%)	169(28.2%)	.88(.56-1.37)	1.10(.64-1.89)	.73
Unknown	72(12.0%)	34(5.7%)	1		1
Presence of fever					
Yes	150(25.0)	100(16.7)	0.68(0.49-0.96)	1.06(.69-1.63)	.78
No	240(40.0)	110(18.3)	1	1	
Presence of cough					
Yes	49(8.2)	66(11.0)	0.31(0.21-0.48)	.47(28-.80)	.005
No	341(56.8)	144(24.0)	1	1	
Presence of diarrhea					
Yes	31(5.2)	46(7.7)	0.31(0.19-0.50)	.46(.25-.86)	.015
No	359(59.8)	164(27.3)	1	1	
Presence of vomiting					
Yes	79(13.2)	73(12.2)	0.47 (0.33-0.70)	.71(.43-1.17)	.18
No	311(51.8)	137(22.8)	1	1	
Presence of edema					
Yes	79(13.2)	79(13.2)	0.42(0.29-0.61)	1.24(.53-2.93)	.62
No	311(51.9)	130(21.7)	1	1	
Child take vit A					
Yes	298(49.7)	146(24.3)	1.42(.98-2.07)	1.20(.76-1.90)	.433

No	92(15.3)	64(10.7)	1	1	
PO antibiotics					
Yes	361(60.2)	179(29.8)	2.16(1.26-3.69)	3.38(1.61-7.08)	.001
No	29(4.8)	31(5.2)	1	1	
Admission category					
New	376(62.8)	164(27.4)	7.33(3.91-13.71)	3.78(1.77-8.07)	.001
Re-admission	14(2.3)	45(7.5)			
Immunization status					
Vaccination for age	343(57.2)	101(16.8)	10.38(5.83-18.47)	6.85(3.68-12.76)	<.001
Partially vaccinated	29(4.8)	54(9.0)	1.64(.82-3.30)	1.26(.60-2.67)	.54
Not vaccinated /unknown	18(3.0)	55(9.2)	1	1	
Weight of the child					
≤ 6.5kg	81(13.5%)	147(24.5%)	.55(.39-.78)	1.08(.70-1.65)	.734
>6.5 kg	129(21.5%)	243(40.5%)	1	1	

Discussion

The study was mainly aimed to indi[illegible]s treatment outcomes of OTP and associated factors with it among children treated f[illegible]m SAM. Accordingly, the overall prevalence of cured, dead, defaulter, and medical transfer were 65.0, 2.0, 16.0, and 17.0 respectively. The result revealed 600 (65%) SAM children admitted to OTP were recovered. This indicates the recovery rate was lower than the sphere standard acceptable range [(30)]. The finding was also lower compared to 76.8 and 80% recovery rates from the study done in OTP Tigiray and Zambia. However, it was comparable to 64.9-62.4% rates from a study done in Wolaita zone(3, 8). The disparities in reports might be due to difference in settings where SAM management was carried out. This study finding is higher than the previous study done in Nigeria 58%. The possible reason for this discrepancy might be the increased Ethiopian Government efforts to improve maternal and child nutrition through a community-based Health Extension Program and variation in the study setting.

The overall defaulter rate in this study inline with study in Tigray 17.5%. This finding is higher as compared to study finding from Wolaita(3, 8). This discrepancy might be due to the increased emphasis to community based therapeutic feeding program. Marasmus was found the predominant form of malnutrition in this study (75.2%), which is in line with the study done at Tigray region (98.4%) and Wolaita(63.4)(3, 8)This may be explained by the fact that marasmus is more common in the age group below two years which is the case in this study in which 70.2% of the study population lies in the age category less-than two years. Regarding death rate according to this study, found that lower proportion of death (2%) this in line with previous findings in wolaita and Tigray region(3, 8). It was also lower than the recommended minimum sphere standard which should be <10%. In this study, the proportion of death was lower this is mainly could be children reach OTP early before developing complications. The other reason may be appropriate management of children such as prescription of routine medication.

Children provided with amoxicillin were 3.38 times more likely to recover as compared to their counterparts. This result was consistent with the finding from North Ethiopia(8). This can be explained by the supportive effect of amoxicillin in the treatment infections and other complications associated with SAM (7). All children might not show clinical signs and symptoms of systematic infections as a result of their low body immunity. According to our study, children who were fully and partially vaccinated had better recovery rate(AOR=6.85, 95% CI: 3.68-12.78)) as compared to those who have been not vaccinated which is almost consistent with a study in Bahirdar Felege Hiwot hospital 4.4 times(17).The probable reasons to this similarity, immunization against microorganism that causes disease can prepare the body immune system, thus helping to fight or prevent infection and an easier way to become immune to a particular disease.

Regarding admission category, the current study revealed that higher odds of recovery were observed among newly admitted children as compared to those who readmitted [AOR=3.78, 95% CI: (1.77-8.07)]. This could be explained as children whose readmitted come with more complication, which ultimately decrease the recovery rate.

Conclusions

Recovery rates in the study area are below the cut of points of the minimum standard sets in humanitarian and disaster prevention (or the sphere standards), it is low as compared to similar studies conducted in different parts of Ethiopia but the death rate was lower than the international standard. Presence of cough comorbidities was statistically significant factors that hinder recovery rate of malnourished children. On the other hand, vaccination and took PO antibiotics were positive indicators for recovery. Attachment of follow-up chart to the individual folder and monitoring of the child progress with the chart also has a greater contribution in improving the recovery of children with severe acute malnutrition in the TFU. Thus, the health care providers should emphasize those SAM cases with comorbidity like cough and readmission case which need strict follow up according to the protocol and increase use of SAM management follow up the chart for all SAM patients. It is also recommended to give community-based health education and counseling for mothers to enhance child immunization.

Abbreviations and Acronyms

EDHS: Ethiopian Demographic Health Serve; IMCI: Integrated Management of Childhood; MOH: Ministry of health; MUAC: Mid Upper Arm Circumference; MAM: Moderate Acute Malnutrition; OTP: Outpatient Therapeutic Program; RUTF: Ready to Use Therapeutic Food; SAM: Severe Acute Malnutrition; TFU: Therapeutic feeding unit; UNICEF: United Nations Children's Fund; WFH: Weight For Height.

Declaration

Ethics approval and consent to participate

Ethical approval was obtained from the research ethics review board of the WU faculty of health science. An official letter of permission was obtained from WU faculty of health science and was submitted to the respective administrative bodies of the Gubalafto Woreda; permission from these administrative bodies was also given. Confidentiality was ensured throughout the research process. All incomplete charts were considered as non-response rate.

Consent for publication

Note applicable.

Availability of data and materials

Data is available and it can be accessed from the corresponding author when asked with the reasonable inquiry.

Competing interests

The authors declare that they have no competing interests.

Funding

None

Authors' contributions

BB, BD, AM, and MW participated in all steps of the study from its commencement to writing. BB, BD, MW, and AM participated in analysis and write-up. All the authors had reviewed and approved the submission of the paper.

Acknowledgments

First of all, we would like to extend our thanks to Woldia University Faculty of Health Sciences, Department of Nursing for providing us the golden opportunity to carry out this study.

Also, we would thank the North Wollo Zone and Gubalafto Wereda Administrative Health Office for their cooperation for giving information related to the general information about the stud area and study population.

Authors' information

All Authors are permanent workers at Woldia University, Faculty of Health Sciences, and Department of Nursing: P.O. Box 400, Woldia, Ethiopia.

References

1. Mengesha MM, Deyessa N, Tegegne BS, Dessie Y. Treatment outcome and factors affecting time to recovery in children with severe acute malnutrition treated at outpatient therapeutic care program. Global health action. 2016;9(1):30704.

2. Munns CF, Shaw N, Kiely M, Specker BL, Thacher TD, Ozono K, et al. Global consensus recommendations on the prevention and management of nutritional rickets. Hormone research in pediatrics. 2016;85(2):83-106.

3. Kabalo MY, Seifu CN. Treatment outcomes of severe acute malnutrition in children treated within the Outpatient Therapeutic Program (OTP) at Wolaita Zone, Southern Ethiopia: a retrospective cross-sectional study. Journal of Health, Population, and Nutrition. 2017;36(1):7.
4. Organization WH, UNICEF. Community-based management of severe acute malnutrition: a joint statement by the World Health Organization, the World Food Programme, the United Nations System Standing Committee on Nutrition and the United Nations Children's Fund. 2007.
5. UNICEF. Evaluation of community management of acute malnutrition (CMAM). Ethiopia: Federal Ministry of Health, Government of Ethiopia and UNICEF Country Office. 2012.
6. Maleta K, Amadi B. Community-based management of acute malnutrition (CMAM) in sub-Saharan Africa: case studies from Ghana, Malawi, and Zambia. Food and nutrition bulletin. 2014;35(2_suppl1): S34-S8.
7. Golden M, Grellety Y. Protocol for the management of severe acute malnutrition. Ethiopia: MOH. 2007.
8. Yebyo HG, Kendall C, Nigusse D, Lemma W. Outpatient therapeutic feeding program outcomes and determinants in treatment of severe acute malnutrition in Tigray, Northern Ethiopia: a retrospective cohort study. Plos One. 2013;8(6):e65840.
9. Organization WH. Guideline: Updates on the management of severe acute malnutrition in infants and children: World Health Organization; 2013.
10. Achadi E, Ahuja A, Bendech MA, Bhutta ZA, De-Regil LM, Fanzo J, et al. Global Nutrition Report: From Promise to Impact: Ending Malnutrition by 2030: International Food Policy Research Institute; 2016.
11. Harvey BM, Eussen SR, Harthoorn LF, Burks AW. Mineral intake and status of cow's milk allergic infants consuming an amino acid-based formula. Journal of pediatric gastroenterology and nutrition. 2017;65(3):346.
12. Lenters LM, Wazny K, Webb P, Ahmed T, Bhutta ZA. Treatment of severe and moderate acute malnutrition in low-and middle-income settings: a systematic review, meta-analysis, and Delphi process. BMC public health. 2013;13(3): S23.
13. Yohannes T, Laelago T, Ayele M, Tamrat T. Mortality and morbidity trends and predictors of mortality in under-five children with severe acute malnutrition in Hadiya zone,

South Ethiopia: a four-year retrospective review of hospital-based records (2012–2015). BMC Nutrition. 2017;3(1):18.

14. UNICEF Wawa. joint child malnutrition estimation. 2017.

15. Dent N, Deconinck H, Golden K, Brown R, Walsh A. Information-sharing to improve learning about community-based management of acute malnutrition (CMAM) and its impact. Food and nutrition bulletin. 2014;35(2_suppl1): S86-S9.

16. Desta K. Survival status and predictors of mortality among children aged 0–59 months with severe acute malnutrition admitted to stabilization center at Sekota Hospital Waghemra Zone. J Nutr Disord Ther. 2015;5:160.

17. Desyibelew HD, Fekadu A, Woldie H. Recovery rate and associated factors of children age 6 to 59 months admitted with severe acute malnutrition at the inpatient unit of Bahir Dar Felege Hiwot Referral hospital therapeutic feeding unite, northwest Ethiopia. PloS one. 2017;12(2):e0171020.

18. Chamois S, Golden M, Grellety Y. Ethiopia Protocol for the management of Severe Acute Malnutrition (2007). 2007.

19. Beer SS, Juarez MD, Vega MW, Canada NL. Pediatric malnutrition: putting the new definition and standards into practice. Nutrition in Clinical Practice. 2015;30(5):609-24.

20. Ahmed AU, Ahmed TU, Uddin MS, Chowdhury MHA, Rahman MH, Hossain MI. The outcome of standardized case management of under-5 children with severe acute malnutrition in three hospitals of Dhaka city in Bangladesh. Bangladesh Journal of Child Health. 2013;37(1):5-13.

21. Chitekwe S, Biadgilign S, Tolla A, Myatt M. Mid-upper-arm circumference based case-detection, admission, and discharging of under-five children in large-scale community-based management of acute malnutrition program in Nigeria. Archives of Public Health. 2018;76(1):19.

22. Mekuria G, Derese T, Hailu G. Treatment outcome and associated factors of severe acute malnutrition among 6–59 months old children in Debre Markos and Finote Selam hospitals, Northwest Ethiopia: a retrospective cohort study. BMC Nutrition. 2017;3(1):42.

23. Chane T, Oljira L, Atomesa E, Agedew E. Treatment outcome and associated factors among under-five children with severe acute malnutrition admitted to therapeutic feeding unit in Woldia hospital, North Ethiopia. Nutrition & Food Sciences. 2014.

24. Dereje N. Determinants of severe acute malnutrition among under-five children in Shashogo Woreda, southern Ethiopia: a community based matched case-control study. J Nutr Food Sci. 2014;4(5):300.
25. Awoke A, Ayana M, Gualu T. Determinants of severe acute malnutrition among under-five children in rural Enebsie Sarmidr District, East Gojjam Zone, North West Ethiopia, 2016. BMC Nutrition. 2018;4(1):4.
26. Chisti MJ, Salam MA, Bardhan PK, Faruque AS, Shahid AS, Shahunja K, et al. Treatment failure and mortality amongst children with severe acute malnutrition presenting with cough or respiratory difficulty and radiological pneumonia. PLoS One. 2015;10(10):e0140327.
27. Admasu A, Tadesse E, Moshago T, Mekonnen N. Survival Status and It's Associated Factors among Under-Five Children Admitted with Complicated Severe Acute Malnutrition in Hospitals of Wolaita Zone, South Ethiopia: Retrospective Cohort Study. 2017.
28. Asres DT, Prasad RP, Ayele TA. Recovery time and associated factors of severe acute malnutrition among children in Bahir Dar city, Northwest Ethiopia: an institution based retrospective cohort study. BMC Nutrition. 2018;4(1):17.
29. Hiwot W/Kidan Ayalkibe treatment Outcomes and Its associated factors among Children with Severe Acute Malnutrition at Therapeutic Feeding Units of Regional Hospitals of Addis Ababa E, 20118.
